# Supplementary material for: A novel genome-wide in vivo screen for metastatic suppressors in human colon cancer identifies the positive WNT-TCF pathway modulators TMED3 and SOX12
Source: EMBO Mol Med. 2014 Jun 11;6(7):882–901. doi: 10.15252/emmm.201303799 (PMC4119353; doi:10.15252/emmm.201303799)
Supplement: Supplementary file 1 — Supplementary Figure S1 [file emmm0006-0882-SD1.pdf]

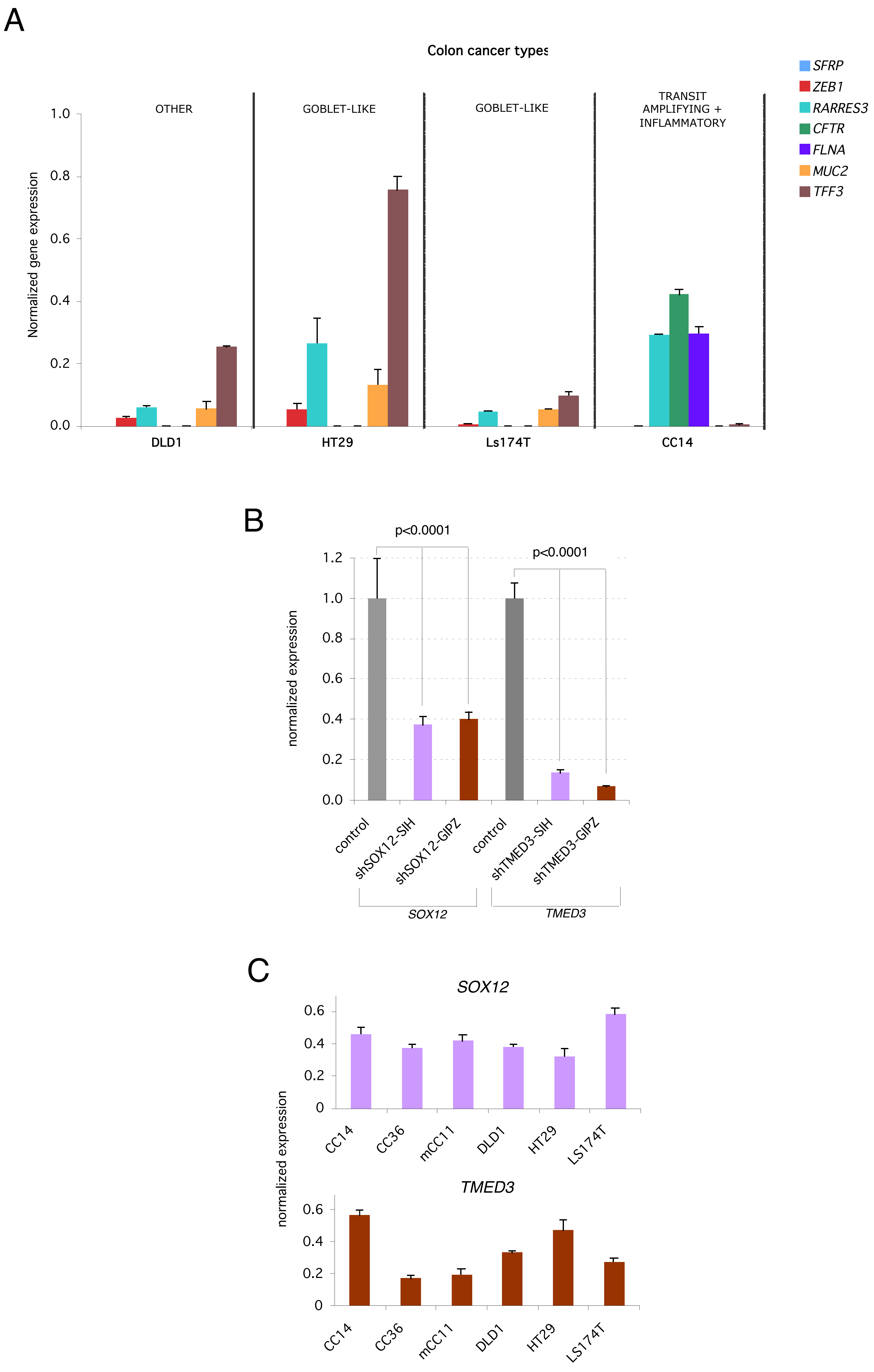

Duquet et al Figure S1

Supplementary Figure S1. Typing colon cancer cells, ShRNA efficiencies and expression of *TMED3* and *SOX12* in colon cancer cells

A) Typing of colon cancer cell lines and primary colon cancers by RT-qPCR of the selected genes according to Sadanandam et al (2013), using the reported primers and conditions. The three cell lines yielded a type in agreement with that reported by Sadanandam et al (2013) and served as internal controls. For easy comparison, normalized ct values by housekeeping genes are shown as fractions where 1 is maximal expression. The top part shows the histogram of the expression values for the genes given in the bottom part.

B) Histograms of RT-qPCR results showing the efficiency of different shRNAs in knocking down their respective targets in the first batch of cells used. Control refers to empty GIPZ vector (see also Fig. 8A).

C) Gene expression histograms of RT-qPCR results showing the presence of *TMED3* and *SOX12* mRNAs in various primary colon cancer cells (CC14, CC36, mCC11) and cell lines (DLD1, HT29, Ls174T). In all cases, y axes show ct levels normalized by those of housekeeping genes (see Materials and Methods).
