# Supplementary material for: A novel genome-wide in vivo screen for metastatic suppressors in human colon cancer identifies the positive WNT-TCF pathway modulators TMED3 and SOX12
Source: EMBO Mol Med. 2014 Jun 11;6(7):882–901. doi: 10.15252/emmm.201303799 (PMC4119353; doi:10.15252/emmm.201303799)
Supplement: Supplementary file 2 — Supplementary Figure S2 [file emmm0006-0882-SD2.pdf]

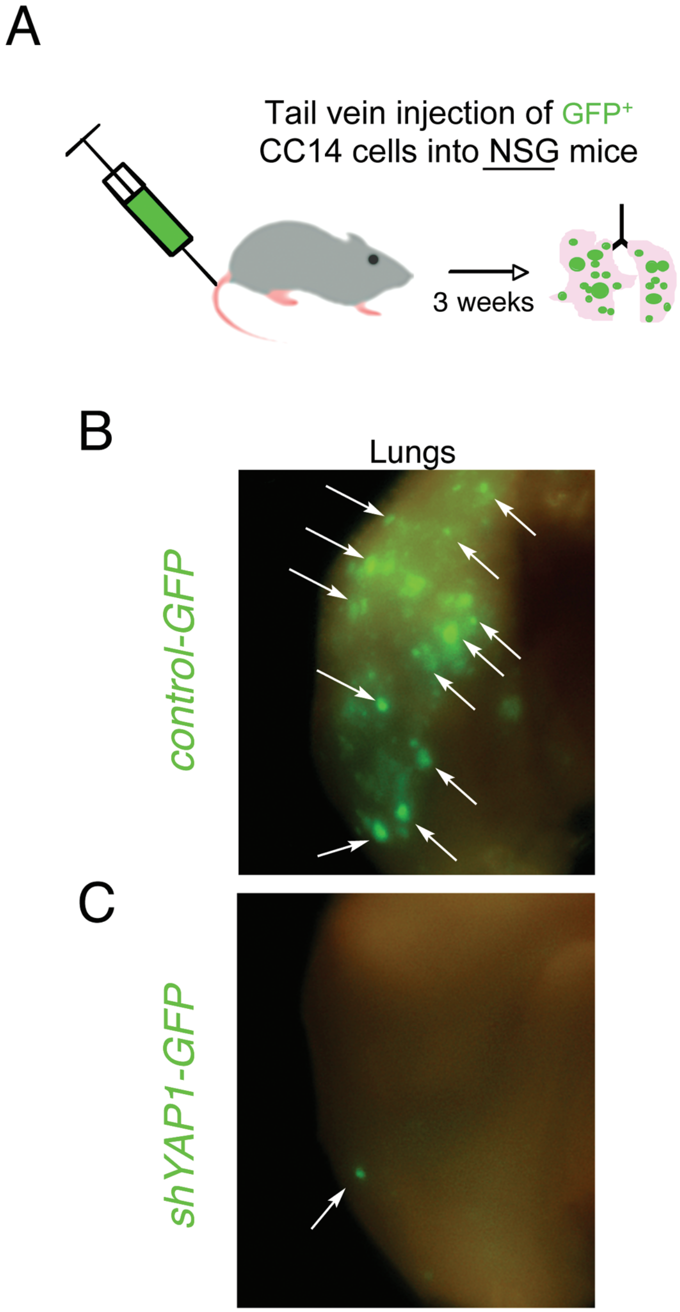

Duquet et al Figure S2

Supplementary Figure S2. Effect of knockdown of *YAP1* on lung metastatic growth in NSG mice.

A) Scheme of tail vein injections into NSG mice (see Fig. 2E).

B) Representative pictures of dissected lung lobes under combined GFP fluorescence illumination and white light. White arrows indicate examples of human colon cancer GFP^+ metastases.
