# Supplementary material for: A novel genome-wide in vivo screen for metastatic suppressors in human colon cancer identifies the positive WNT-TCF pathway modulators TMED3 and SOX12
Source: EMBO Mol Med. 2014 Jun 11;6(7):882–901. doi: 10.15252/emmm.201303799 (PMC4119353; doi:10.15252/emmm.201303799)
Supplement: Supplementary file 3 — Supplementary Figure S3 [file emmm0006-0882-SD3.pdf]

A

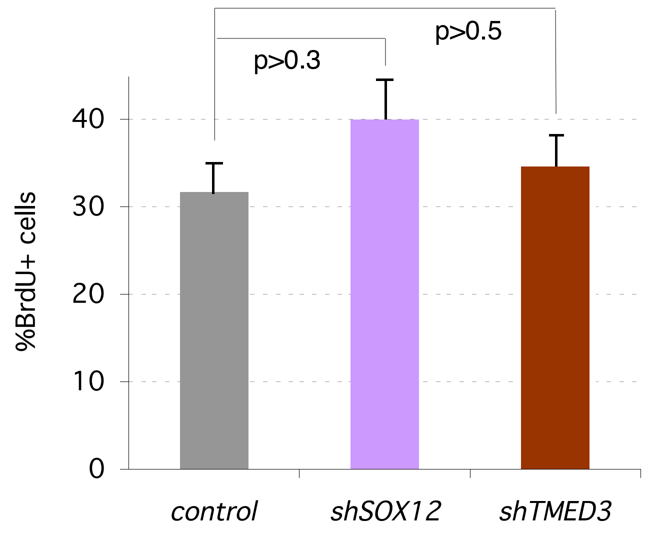


B

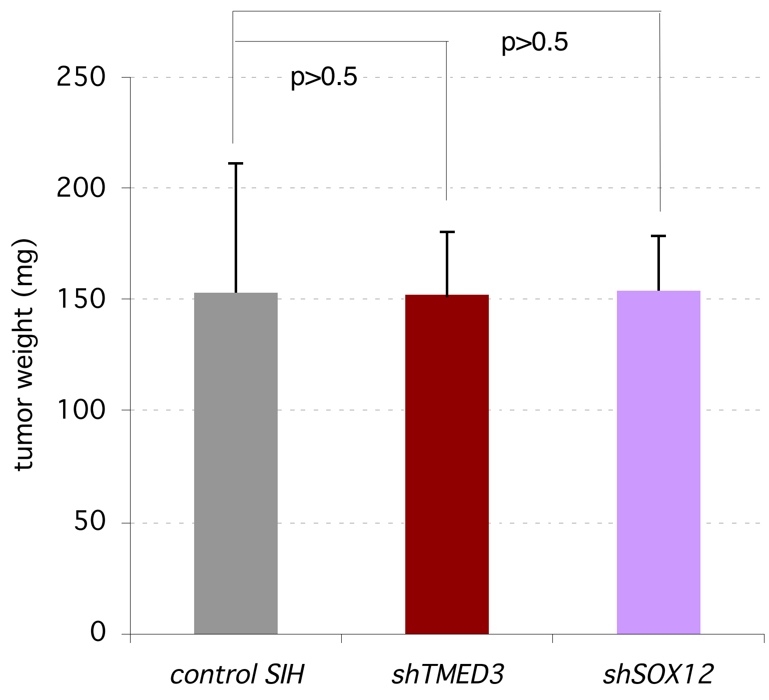


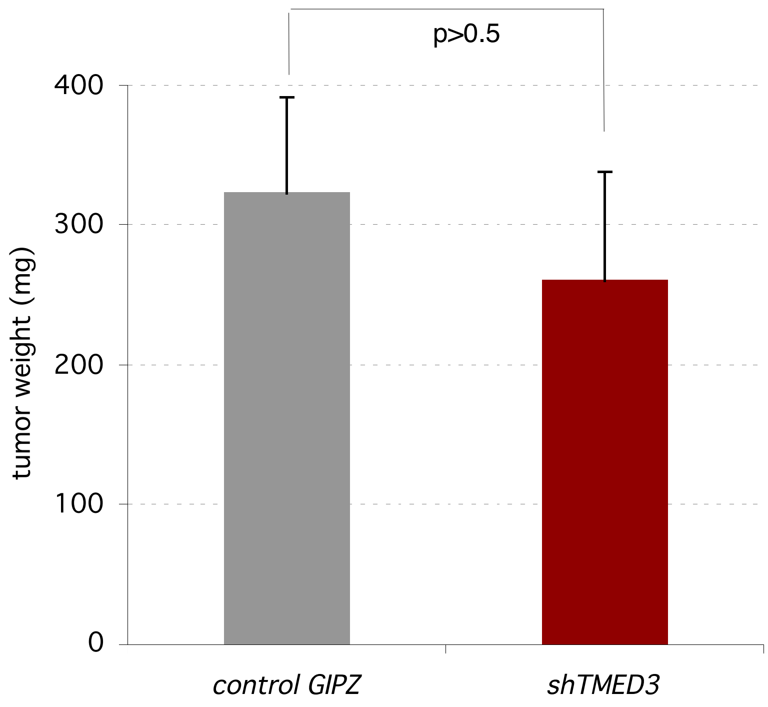


Duquet et al Figure S3

**Supplementary Figure S3. Effects of knockdown of SOX12 or TMED3 on cell proliferation in vitro and tumor growth in vivo.**

A) Histogram of the quantification of BrdU incorporation in CC14 cells under the indicated conditions in vitro. No significant differences were observed.

B) Histograms of two separate experiments measuring the tumor volumes of control (parental SIH or GIPZ lentivectors), and shSOX12 or shTMED3 in the matching vectors as shown. No differences were observed in tumor growth in homogeneous grafts.
