# Supplementary material for: A novel genome-wide in vivo screen for metastatic suppressors in human colon cancer identifies the positive WNT-TCF pathway modulators TMED3 and SOX12
Source: EMBO Mol Med. 2014 Jun 11;6(7):882–901. doi: 10.15252/emmm.201303799 (PMC4119353; doi:10.15252/emmm.201303799)
Supplement: Supplementary file 4 — Supplementary Figure S4 [file emmm0006-0882-SD4.pdf]

A

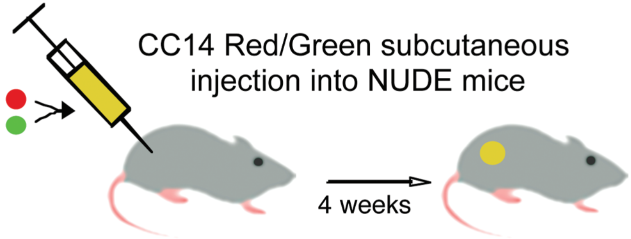

B

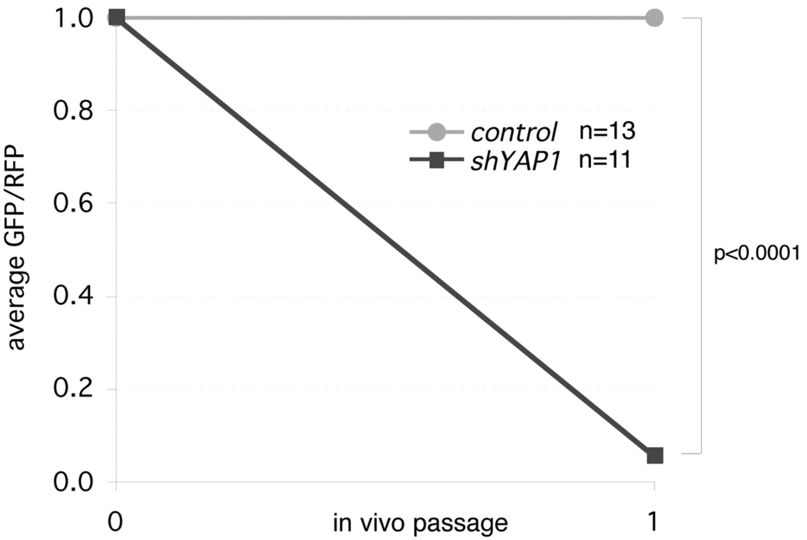

Duquet et al Figure S4

Supplementary Figure S4. YAP1 is required for efficient competition in vivo.

A) Scheme of the in vivo red/green competition assay used (see Fig. 3).

B) Quantification of the GFP^+/RFP^+ cell ratios, as determined by FACS analyses after cell dissociation, of control RFP^+ cells combined with either control GFP^+ or *shYAP1* GFP^+ cells over one passage in vivo.
