# Supplementary material for: A novel genome-wide in vivo screen for metastatic suppressors in human colon cancer identifies the positive WNT-TCF pathway modulators TMED3 and SOX12
Source: EMBO Mol Med. 2014 Jun 11;6(7):882–901. doi: 10.15252/emmm.201303799 (PMC4119353; doi:10.15252/emmm.201303799)
Supplement: Supplementary file 5 — Supplementary Figure S5 [file emmm0006-0882-SD5.pdf]

Nude

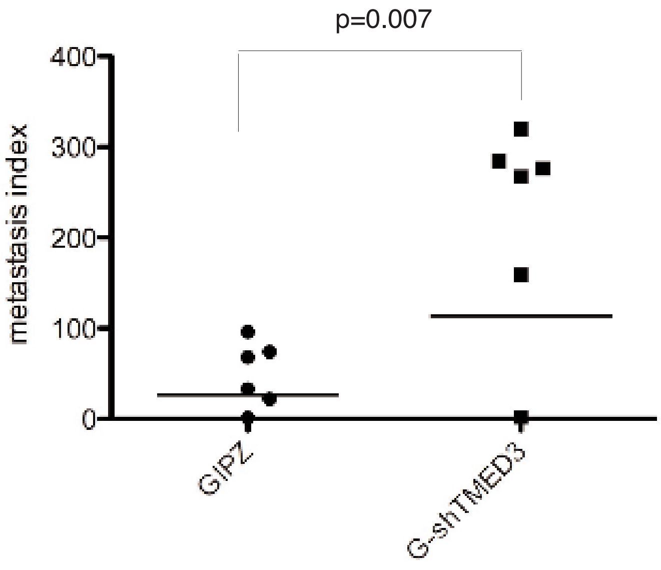

NSG

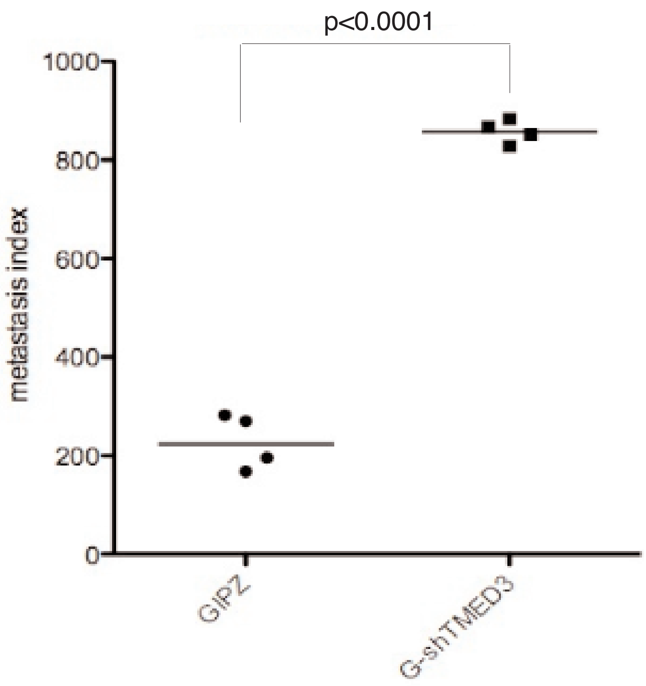

Duquet et al Figure S5

Supplementary Figure S5. Quantification of metastatic indexes in Nude and NSG mice subcutaneously grafted with control or TMED3-knockdown cells.

Scatter plots of the number of metastases detected in experiments performed with control GIPZ vector or GIPZ-*shTMED3* either in Nude or in NSG mice. The y axes represent the metastatic index, which is the number of metastases divided by the tumor weigth (see Fig. 4).
