# Supplementary material for: A novel genome-wide in vivo screen for metastatic suppressors in human colon cancer identifies the positive WNT-TCF pathway modulators TMED3 and SOX12
Source: EMBO Mol Med. 2014 Jun 11;6(7):882–901. doi: 10.15252/emmm.201303799 (PMC4119353; doi:10.15252/emmm.201303799)
Supplement: Supplementary file 6 — Supplementary Figure S6 [file emmm0006-0882-SD6.pdf]

A

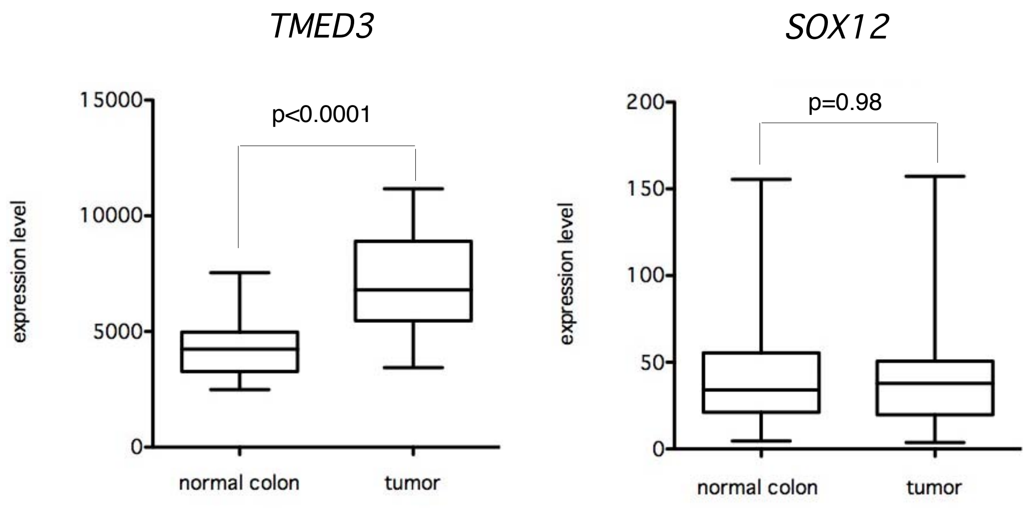

B

		TMED3	*SOX12*
TMN1	CC76	1.7	0.8
TMN2	CC72	0.8	1.4
	CC74	1.2	8.6
	CC79	1.5	1.1
	CC80	1.6	0.02
TMN3	CC12	0.6	1.0
	CC75	1.6	0.9
	CC77	3.5	0.9
	CC78	6.3	0.7
TMN4	CC10	1.7	2.2
	CC70	2.3	1.1
	CC73	1.1	1.0
	CC60	0.3	0.9
Liver mets	mCC1	2.3	1.4
	mCC2	3.9	3.1

Duquet et al Figure S6

Supplementary Figure S6. *TMED3*, but not *SOX12*, expression is enhanced in colon cancers versus normal colon.

A) Box plots showing the expression level of *SOX12* and *TMED3* genes in normal and colon tumors derived from the public dataset GSE10950 (see Fig. S7).

B) RT-qPCR heat map table of the expression levels of *TMED3* and *SOX12* mRNAs as determined by RT-qPCR. Samples are those described in Varnat et al. (2009) plus new samples from the operating room described in Fig. S8. Results are expressed as fold expression over control (using paired normal colon).
