# Supplementary material for: A novel genome-wide in vivo screen for metastatic suppressors in human colon cancer identifies the positive WNT-TCF pathway modulators TMED3 and SOX12
Source: EMBO Mol Med. 2014 Jun 11;6(7):882–901. doi: 10.15252/emmm.201303799 (PMC4119353; doi:10.15252/emmm.201303799)
Supplement: Supplementary file 7 — Supplementary Figure S7 [file emmm0006-0882-SD7.pdf]

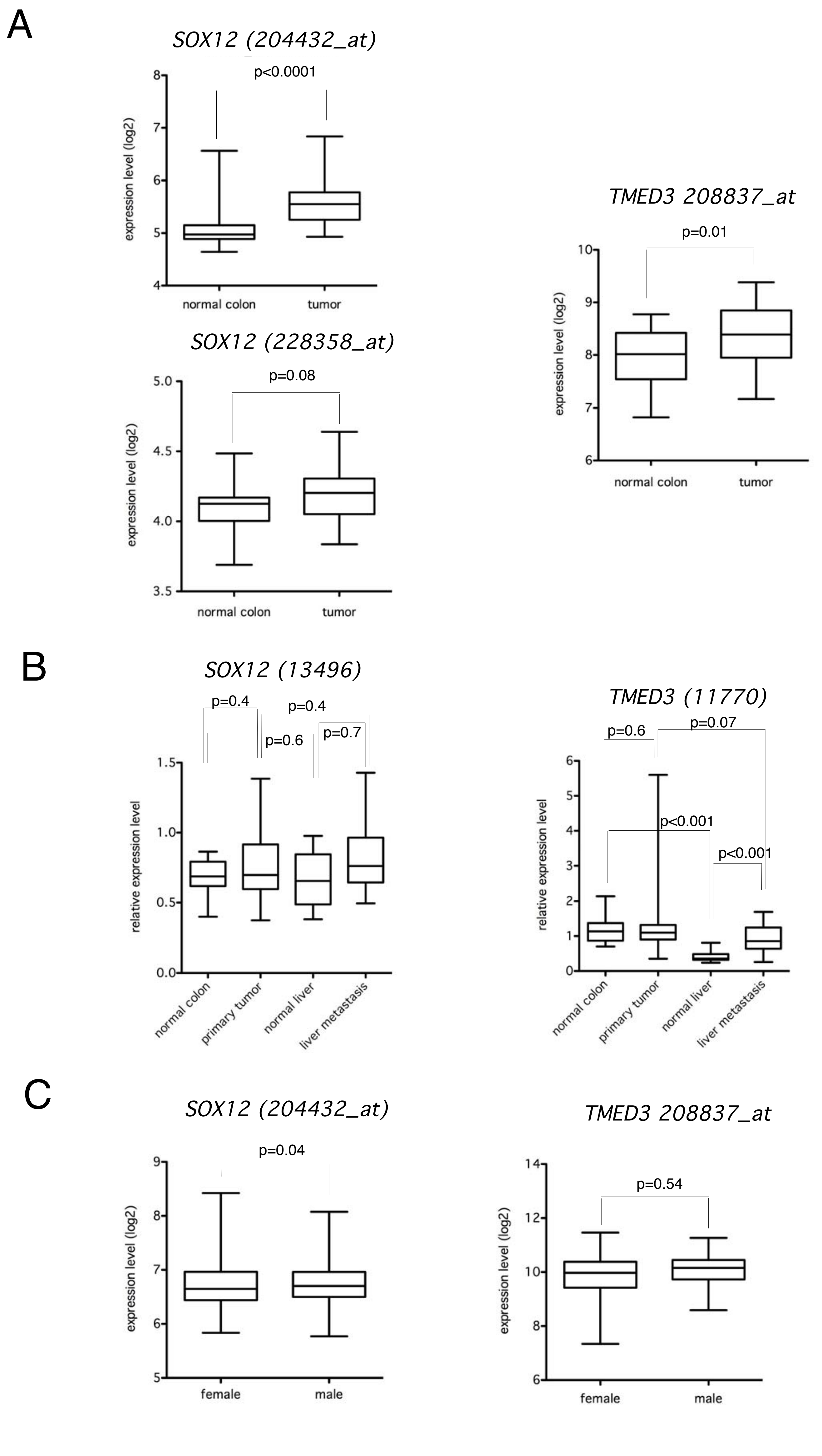

Duquet et al Figure S7

Supplementary Figure S7. Data mining of public databases for the expression of SOX12 and TMED3.

A) Box plots showing the expression level of *SOX12* and *TMED3* genes in normal colon and colon cancers derived from the public dataset GSE23878. Two probes were available for *SOX12*: probe 204432_at (upper panel) and probe 228358_at (lower panel)

B) Box plots showing the expression level of *SOX12* and *TMED3* genes in paired normal colon, normal liver, colon cancers and liver cancer metastases derived from the public dataset GSE6988. Probe 13496 was used *SOX12* for and 11770 for *TMED3*.

C) Box plots showing lack of major differences in expression levels of *SOX12* or *TMED3* associated with gender.
