# Supplementary material for: A novel genome-wide in vivo screen for metastatic suppressors in human colon cancer identifies the positive WNT-TCF pathway modulators TMED3 and SOX12
Source: EMBO Mol Med. 2014 Jun 11;6(7):882–901. doi: 10.15252/emmm.201303799 (PMC4119353; doi:10.15252/emmm.201303799)
Supplement: Supplementary file 8 — Supplementary Figure S8 [file emmm0006-0882-SD8.pdf]

	Age	Gender	Location	TNM	stage
CC76	75	male	right colon	T2N0M0	1
CC72	83	male	right colon	T3N0M0	2
CC79	59	female	left colon	T3N0M0	2
CC80	63	male	left colon	T3N0M0	2
CC12	82	male	rectum	T3N1M0	3
CC75	72	female	right colon	T4N2M0	3
CC77	88	female	rectum	T3N1M0	3
CC78	71	male	left colon	T3N2M0	3
CC10	74	female	rectum	T3N2M1	4
CC70	49	female	left colon	T4N2M1	4
CC73	54	male	na	T4N2M1	4
CC60	66	male	sigmoid/left colon	T3N2M1	4
mCC1	66	female	liver met	T0N1M1	4
mCC2	64	male	liver met	T3N2M1	4

Duquet et al Figure S8

Supplementary Figure S8. Human tumor data.

Medical data of primary colon tumor samples used in this study not described in Varnat et al. (2009; 2010).

na = data not available.
