# Supplementary material for: A novel genome-wide in vivo screen for metastatic suppressors in human colon cancer identifies the positive WNT-TCF pathway modulators TMED3 and SOX12
Source: EMBO Mol Med. 2014 Jun 11;6(7):882–901. doi: 10.15252/emmm.201303799 (PMC4119353; doi:10.15252/emmm.201303799)
Supplement: Supplementary file 9 — Supplementary Figure S9 [file emmm0006-0882-SD9.pdf]

A

	shSOX12	*shTMED3*	*dnTCF4*
AXIN2	0.4	0.4	0.3
EPHB2	0.6	0.3	0.3
LGR5	0.5	2.3	0.1
P21	0.6	1.6	2.9
SOX4	5.3	4.5	2.1
DKK1	1.7	2.8	3.3
B-CAT	0.8	0.9	1.0
SOX12	0.3		
TMED3		0.3	

HT29

B

CC14

	shSOX12	*shSOX12+[illegible]CAT*	*shTMED3*	*shTMED3+[illegible]CAT*
EPHB2	0.1	1.9	0.1	2.6
ASCL2	0.1	5.9	0.6	5.2
LGR5	0.7	4.3	0.2	3.9
AXIN2	0.9	2.0	0.9	1.6

HT29

EPHB2	0.5	23.7	0.1	19.1
ASCL2	0.8	8.2	0.4	5.1
LGR5	0.4	7.8	1.2	8.3
AXIN2	0.3	10.7	0.2	7.9

Duquet et al Figure S9

Supplementary Figure S9. Changes in WNT-TCF target gene expression levels in HT29 cells after knock-down of *TMED3* or *SOX12* and rescue by co-expressed active βCATENIN.

A) Heat map of changes in gene expression after knock-down of TMED3 or SOX12 or expression of dnTCF. Changes are expressed as ratios over vector-alone transduced controls, after normalization with housekeeping genes. Red boxes indicate overexpression and blue boxes underexpression. The third column shows the changes obtained with dnTCF as control. The two lower rows shows the level of KD of the specific targets compared with control-vector only expressing cells (which was equated to 1).

B) Heat map of changes after knock-down of TMED3 or SOX12 as in (A) and (Fig. 8A) (left column) and after co-expression of active N'ΔβCATENIN. Values are all expressed as ratios over vector-alone transduced controls, after normalization with housekeeping genes. Red boxes indicate overexpression and blue boxes underexpression over controls in the left column. On the right column, however, red boxes refer to overexpression (rescue) over the values on the left columns.
