# Supplementary material for: A novel genome-wide in vivo screen for metastatic suppressors in human colon cancer identifies the positive WNT-TCF pathway modulators TMED3 and SOX12
Source: EMBO Mol Med. 2014 Jun 11;6(7):882–901. doi: 10.15252/emmm.201303799 (PMC4119353; doi:10.15252/emmm.201303799)
Supplement: Supplementary file 10 — Supplementary Figure S10 [file emmm0006-0882-SD10.pdf]

	**FORWARD**	**REVERSE**
	5'->3'	5'->3'
TBP	TGCACAGGAGCCAAGAGTGAA	CACATCACAGCTCCCCACCA
HMBS	AAGTGCGAGCCAAGGACCAG	TTACGAGCAGTGATGCCTACCAAC
β-ACTIN	TGGAGAAAATCTGGCACCACACC	GATGGGCACAGTGTGGGTGACCC
GLI1	GGGCACCATCCATTTCTACA	CCTGCATTGCCAGTCATTT
GLI2	AGCAGCAGCAACTGTCTGAG	CACATGAGCCGTGTCCAG
PTCH1	CCACAGAAGCGCTCCTACA	CTGTAATTTCGCCCCTTCC
ASCL2	GCGTTCCGCCTACTCGT	GGCTTCCGGGGCTGAG
LGR5	GGAGCATTCACTGGCCTTTA	CTGGACGGGGATTTCTGTTA
OLFM4	GGACTTCGAGCTGATCAAGG	CGACAGGGGTGTTTTGATCT
AXIN2	CTCCTTATCGTGTGGGCAGT	CCAACTCCAGCTTCAGCTTT
EPHB2	GCCTGTGTGCACTTATGCAG	CTTGGTCCGTAGCCAGTTTGT
P21	GACTCTCAGGGTCGAAAACG	AAGATGTAGAGCGGGCCTTT
β-CAT	ATGATGCAGAACTTGCCACA	CATCTGAGGAGAACGCATGA
NANOG/P8	GGATGGTCTCGATCTCCTGA	CGGCAGCCAAGGTTATTAAA
E-CAD	GGATGTGCTGGATGTGAATG	CTCAAAATCCTCCCTGTCCA
SNAIL1	GAAAGGCCTTCAACTGCAAA	TGACATCTGAGTGGGTCTGG
SOX12	GCCTCCCCGACACCGTCGGA	ATCCAGGGCGCTGCAGTCCA
TMED3	CCTCCCATTCTCCCAGACT	CAATCGTCTCGCCAACAGAC
C-MYC	TGGTCTTCCCCTACCCTCT	GATCCAGACTCTGACCTTTT
CD44	AAGGTGGAGCAAACACAACC	AGCTTTTTCTTCTGCCCACA
YAP1	GCAGTTGGGAGCTGTTTCTC	GCCATGTTGTTGTCTGATCG

Duquet et al Figure S10

Supplementary Figure S10. PCR primer sequences.

Table of primers used for qPCR as described in the text.
